# Impact of dispersive patch electrode positioning on safety and efficacy of radiofrequency catheter ablation

**DOI:** 10.1093/europace/euae285

**Published:** 2024-11-25

**Authors:** Piotr Futyma, Łukasz Zarębski, William H Sauer

**Affiliations:** University of Rzeszów and Clinical Electrophysiology, St. Joseph’s Heart Rhythm Center, Anny Jagiellonki 17, Rzeszów 35-623, Poland; University of Rzeszów and Clinical Electrophysiology, St. Joseph’s Heart Rhythm Center, Anny Jagiellonki 17, Rzeszów 35-623, Poland; Cardiac Arrhythmia Service, Brigham and Women’s Hospital, Harvard Medical School, Boston, MA, USA

**Keywords:** Dispersive patch electrode, Radiofrequency ablation, Radiofrequency current flow, Impedance

## Abstract

Safe and efficient radiofrequency catheter ablation depends significantly on the proper placement of dispersive patch electrodes (DPEs), on the skin. This viewpoint describes the role of DPE positioning in optimizing lesion creation and reducing the risk of complications. Incorrect DPE placement can lead to suboptimal energy delivery, prolonging the procedure and/or increasing the risk of adverse events, such as steam pops and potentially fatal atrio-oesophageal fistula. Despite its importance, there is no consensus on the optimal positioning of DPE, and current studies require further improvement in predictive modelling.

## Graphical Abstract

**Graphical Abstract:**
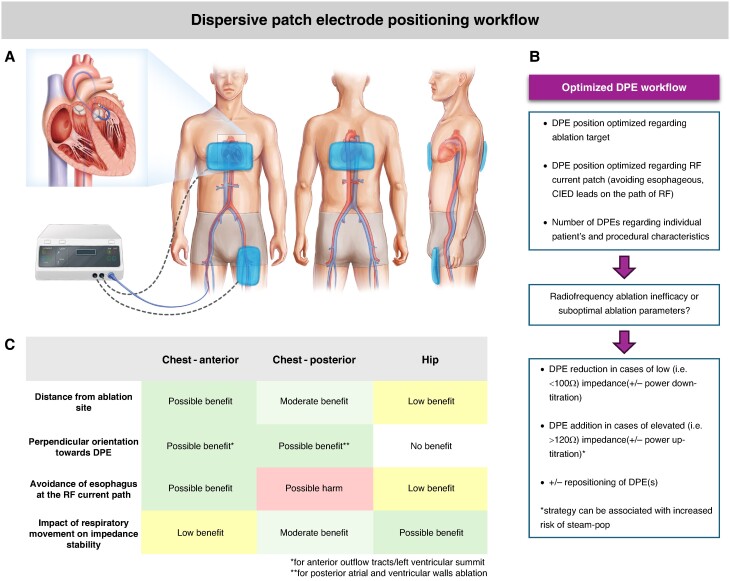

## Introduction

Despite significant evolution in ablation techniques in recent years, some arrhythmias, such as atrial fibrillation or ventricular tachycardia, may sometimes require extensive ablation lesion sets to achieve clinical success.^1^ However, such extensive ablative therapy can accumulate radiofrequency (RF) energy not only in specific cardiac regions but also in extra-cardiac areas, and injuries in these regions have been reported.^2^ During standard monopolar RF catheter ablation (RFCA), the alternating electrical current flows between the catheter tip and a dispersive patch electrode (DPE) attached to the patient’s skin. In a perfect scenario, a discrepancy between the small surface area of the ablation catheter tip positioned close to the ablation target and large surface area of skin DPE results in homogeneous lesion formation within the myocardial tissue. However, the entire monopolar RF ablation system is highly dependent from the relationship between ablation catheter tip and dispersive electrodes, as both components of the RF circuit provide critical impact on alternating RF current flow within the circuit.^3^ While broad spectrum of parameters associated with ablation catheter tip has been tested, little information is available regarding the influence of the DPE placement, as a component within the RF circuit. In addition, the manufacturers of generators provide few or no recommendations concerning the positioning of DPE (*Table 1*). This viewpoint article focuses specifically on the impact of the DPE position on the efficacy and safety of RFCA.

**Table 1:** Recommendations regarding the position of dispersive electrodes during radiofrequency catheter ablation according to the instructions for the use of the most common radiofrequency generators

Radiofrequency generator	Special recommendations
Ampere™	No recommendations
nGen™	In cases of elevated impedance: Improve the contact of the indifferent electrode with the patient’s skin or use a different indifferent electrode. Consider applying an indifferent electrode to a different place on the patient’s body to avoid oil, hair, and dirt that increase the impedance.
Smartablate™	No recommendations
Stockert EP Shuttle™	For optimal performance, the indifferent electrode should be applied close to the operating field.

## Contact

**Figure 1:**
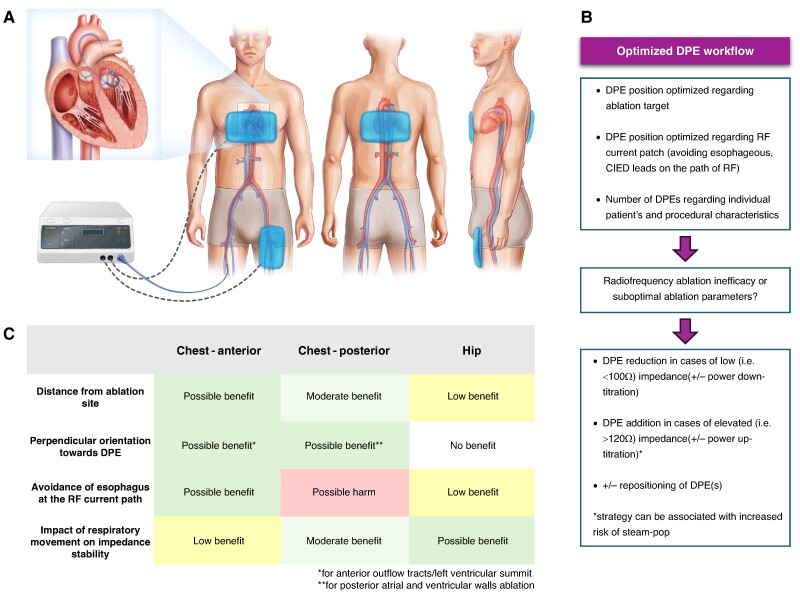
(*A*) Schematic options of dispersive patch positioning during radiofrequency catheter ablation and their relationship with the heart. (*B*) Flowchart demonstrating an optimized DPE workflow during radiofrequency catheter ablation. (*C*) Table comparing pros and cons of different DPE positions during radiofrequency catheter ablation. CIED, cardiac implantable electronic device; DPE, dispersive patch electrode; RF, radiofrequency.

Dispersive patches, also known as grounding pads, are placed on the patient’s body to complete the electrical circuit and ensure the safe dispersion of the electrical energy (*Figure 1A*). Proper positioning of DPE is necessary to optimize lesion creation and minimize RF current density at any site in contact with the skin. Proper skin preparation, including cleaning and shaving, if necessary, helps to ensure contact, minimize resistance, and improve the dispersion of RF electrical energy. Consistent adhesion of the DPE to the skin is essential to maintain consistent contact throughout the procedure and avoid skin injury.

When the dispersive patches are placed incorrectly, it can lead to suboptimal lesion formation and potentially increase the risk of adverse events:

- Elevated impedance caused by inappropriate DPE placement will lead to lower current delivery at a programmed fix power setting. This may cause a need for additional RF lesions at the targeted area and may increase risk caused by prolonged application time.
- Inadequate patch adhesion can lead to RF heating at regions where there is patch contact, occasionally resulting in thermal skin burns.

Factors that can impact DPE-skin contact include the following:

- Type of patch (rubber/multiple use DPE vs. single-use DPE; medium/large area DPE vs. small area DPE)
- Skin surface (presence of hair, skin conditioners, and sweat)

Single-use DPEs are thought to be more likely to provide stable contact with a patient’s skin. However, some types of reusable electrodes are preferred by some for the large area of contact they provide, resulting in lower system impedance.^4^

## Distance

The distance between the DPE and the ablation site is another important factor that affects the current pathway and distribution of energy within the treated cardiac tissue. If the distance is too large, the electrical energy may encounter higher impedance and may result in ineffective heating of targeted tissue.^5^ Conversely, too short distance between the tip of ablation catheter and DPE will result in increased RF current density alternating within the ablation lesion and possible overshooting. The distance between the ablation catheter tip and DPE has a direct impact on baseline impedance, and its elevated value can limit RF current density across the ablation target. Moreover, elevated impedance may change the actual power output of the RF generator, causing the discrepancies between selected and delivered power.^6^

The following factors should be taken into account regarding DPE distance from the ablation target. Placing DPE on relatively large, flat areas of the body with good skin contact, such as the anterior chest wall or upper thigh, may be desired. The positioning should also take into account patient characteristics and individual anatomical and clinical considerations. For example, anaesthesiologic guidelines suggest repositioning the dispersive electrode in patients with implanted cardioverter defibrillator during electrosurgery to avoid electromagnetic interference.^7^

It is worth noting that the development of novel ablation catheters has aimed to mitigate the impact of dispersive patch positioning. For instance, some catheters incorporate multiple electrodes, allowing for simultaneous energy delivery and reducing the reliance on dispersive patches. These advancements can help optimize lesion formation and potentially reduce the sensitivity to dispersive patch position.

## Radiofrequency current flow simulations

Recent simulations of different DPE locations^5,8^ provide some new information about the biophysics of RF energy distribution. Some limitations of these *in silico* models, however, should be acknowledged. Firstly, cardiac lesion depths are calculated without impact on extra-cardiac structures. Secondly, impact of heat sink effect, from aorta and coronary arteries, is not addressed.^9^ Thirdly, impact of cumulative thermal injury of the oesophagus from multiple sequential and overlapping RF applications is lacking. Fourthly, fluctuations in oesophageal resistivity can lead to higher current density in the oesophageal region.^10^ Lastly, the conductivity and resistivity of lungs (of note, different for inspiration and expiration phase) acknowledged in *in silico* models are different from some literature data,^11^ while conductivity and resistivity of other extra-cardiac structures (aortic wall and oesophagus) were not addressed in the simulations. Because of the fact that atrio-oesophageal fistula formation is multifactorial, available *in silico* models may not provide a definitive estimation of RF current pathways in view of DPE positioning. Additionally, compression of a posterior DPE to the patient’s body can impact RF current flow characteristics. Patients with low body mass index can be particularly prone to fluctuations associated with DPE positioning. Altering the RF current vector using DPE repositioning not only can have an impact on cardiac lesion depth^12^ but can also reduce the cumulative effect of power transmitted to extra-cardiac torso areas, as demonstrated in other recent *in silico* model.^13^

## Unresolved issues

A number of DPEs participating in the RF circuit and their distance from the ablation target have an impact on baseline impedance and subsequent electrical current density during RFCA.^14^ Given this, ablation characteristics on homogeneous myocardial tissue are relatively well understood. In contrary, little is known regarding thermal effects and how variations in DPE placement and number might influence thermal injury or lesion formation in more complex or heterogeneous tissues.

The temperature-controlled RF delivery may be less influenced by DPE positioning. However, it is important to note that while temperature-controlled RF can sometimes sufficiently balance the amount RF delivery (in contrast to the power-controlled mode), it is unable to influence the vector of alternating RF current path between ablation catheter tip and the DPE. Secondary, if the thermistors in the ablation catheter tip are usually located distally and if the DPE is placed posteriorly to the curve of ablation catheter, the temperature measurements may be underestimated, potentially leading to overheating at the very proximal part of the distal tip,^15^ but more clinical data are needed.

In conclusion, the position of dispersive patches during catheter RF ablation is an underappreciated but critical factor that affects safe and effective lesion formation. Proper placement, adequate contact area, and appropriate distance from the ablation site are essential to ensure effective energy delivery and minimize complications.

## Data Availability

No new data were generated or analysed in support of this research.
